# Management of pain induced by exercise and mobilization during physical therapy programs: views of patients and care providers

**DOI:** 10.1186/1471-2474-12-172

**Published:** 2011-07-22

**Authors:** Sophie Alami, Dominique Desjeux, Marie Martine Lefèvre-Colau, Anne Sophie Boisgard, Eric Boccard, François Rannou, Serge Poiraudeau

**Affiliations:** 1 Department of Social Sciences, Université Paris Descartes; Interlis, Paris, France; 2 Department of Social Sciences, Université Paris Descartes, Paris, France; 3 AP-HP, Service de Médecine Physique et de Réadaptation Hôpital Corentin Celton; Université Paris Descartes, Paris, France; 4 Bristol-Myers Squibb, Paris, France; 5 AP-HP, Service de rééducation et réadaptation de l'appareil locomoteur et des pathologies du rachis Hôpital Cochin; Université Paris Descartes; INSERM IFR 25 Handicap, Paris, France

## Abstract

**Background:**

The expectations of patients for managing pain induced by exercise and mobilization (PIEM) have seldom been investigated. We identified the views of patients and care providers regarding pain management induced by exercise and mobilization during physical therapy programs.

**Methods:**

We performed a qualitative study based on semi-structured interviews with a stratified sample of 12 patients (7 women) and 14 care providers (6 women): 4 general practitioners [GPs], 1 rheumatologist, 1 physical medicine physician, 1 geriatrician, 2 orthopedic surgeons, and 5 physical therapists.

**Results:**

Patients and care providers have differing views on PIEM in the overall management of the state of disease. Patients' descriptions of PIEM were polymorphic, and they experienced it as decreased health-related quality of life. The impact of PIEM was complex, and patient views were sometimes ambivalent, ranging from denial of symptoms to discontinuation of therapy. Care providers agreed that PIEM is generally not integrated in management strategies. Care providers more often emphasized the positive and less often the negative dimensions of PIEM than did patients. However, the consequences of PIEM cited included worsened patient clinical condition, fears about physical therapy, rejection of the physical therapist and refusal of care. PIEM follow-up is not optimal and is characterized by poor transmission of information. Patients expected education on how better to prevent stress and anxiety generated by pain, education on mobilization, and adaptations of physical therapy programs according to pain intensity.

**Conclusion:**

PIEM management could be optimized by alerting care providers to the situation, improving communication among care providers, and providing education to patients and care providers.

## Background

Society must be prepared for an aging world with an increasing demand for high-quality standards for health-related quality of life (HRQoL). Hence, handicap, particularly involving disability and restricted participation, is becoming important in connection with preventing and treating disease, and populations in developed countries are demanding increased life expectancy without disability, which is a major public health strategy [1-3].

The prevalence of chronic painful conditions, responsible for limiting mobility and restricting participation, is increasing in aging developed countries [4-10]. Many therapeutic programs proposed for painful conditions consist of physical therapy programs with mobilization, and about 70% of outpatients and inpatients are referred to physical therapy programs for painful conditions (mainly neck and low-back pain, lower-limb osteoarthritis, sports injuries, total joint replacement, upper-limb musculoskeletal disorders, inflammatory arthritis) [11-14]. The exercise and mobilization techniques of physical therapy include aerobic training, specific muscular strength exercises, active and passive mobilization, and proprioceptive techniques.

Although these programs have been shown to reduce pain in many mid- and long-term clinical situations [15], they may provoke pain during the execution of exercise or mobilization, thus leading patients to have less compliance with and less confidence in these programs [16,17]. Surprisingly, the management of pain induced by exercise and mobilization (PIEM) during physical therapy programs has received little attention until now.

PIEM is part of a more general problem that can be considered pain induced by care, the main components being pain induced by physical examination, administered personal care, and treatments or studies [18,19].

The patient point of view regarding health status and treatments has become important in decision-making procedures and has been considered a possible criterion standard to assess treatment efficacy [20,21]. Results of several surveys conducted in primary or secondary care situations suggested that patients perceived some painful chronic situations, such as knee osteoarthritis, to be more disabling than hypertension, diabetes mellitus and heart diseases; whereas physicians considered the latter three conditions the most important chronic disabling conditions [22]. Patients with rheumatoid arthritis or osteoarthritis, healthy professionals, and healthy control subjects do not agree on the importance of disabilities [23,24].

Addressing these discrepancies between patients and physicians in defining the importance of an illness associated with substantial decreases in HRQoL should lead to a paradigm shift toward a more patient-centered approach. Taking into account patient priorities and expectations may lead to a better understanding of what is important to patients [25-27].

Despite differences between patients and their physicians in assessing what is important in health and symptom status, the views of patients and practitioners concerning the management of PIEM have been seldom studied. Qualitative research is probably the best way to understand patients' needs and contexts and could improve therapeutic strategies and their assessments, as well as adherence to treatments [28-30]. We aimed to assess patient and care provider views concerning PIEM management by a qualitative approach with semi-structured interviews.

## Methods

### Ethics Statement

The local ethics committee is « le comité de protection des personnes Paris centre, groupe hospitalier Cochin-Broca-Hôtel Dieu ». The survey was conducted in compliance with the Good Clinical Practices and Declaration of Helsinki Principles protocol. In accordance with French national law (loi Huriet), formal approval by an ethical committee is not required for this kind of project (observational study with anonymous data). All patients agreed to participate and gave their written informed consent to be in the study.

### Qualitative interview study

This was a qualitative interview study of patients and care providers performed according to guidelines for inductive qualitative research [31-33].

Semi-structured interviews with both patients and care providers were conducted to explore patient and care provider views about PIEM and pain management. Individual behaviours (attitudes and practices), personal feelings and interpretations, social interactions and material backgrounds were specifically examined throughout the patients' therapeutic journey, to allow for a deep understanding of patient and care provider expectations.

#### Sample

A heterogeneous sample of 12 patients and 14 care providers was selected. The sample selection was based on non-probability judgment sampling, assuring both relevance to the subject and diversity of the members selected. The sample size was determined on the basis of the principle of saturation and maximum variety sampling [31].

Saturation was only a guiding principle. This concept can provide a sample size only contingently (i.e., contingent on data collection and analysis, as per Glaser and Strauss^31^). Therefore, we relied on the professional experience and competency of the research team to determine sample size. With the expertise of the social science researchers, saturation occurs within the first 10 interviews depending on the subject studied and the heterogeneity of the group (about 5 to 15). Moreover, the main data codes were developed after about 5 or 6 interviews (in-depth interviews). We also relied on the expertise of the physician members of the research team to determine the criteria of diversification and, thus, the sample size.

Another guiding principle of our sampling procedure was to explore diversity, and we sought maximum variety in sampling as well. For care providers, we selected a group of 14 participants, with experience in acute or chronic pain management at various points of the therapeutic course, from 2 different categories: physicians (9) and physical therapists (5). We aimed for diversification of points of views and care practices by including a variety of professionals from 7 medical specialties and health care professions (4 general practitioners [GPs], 1 rheumatologist, 1 physical medicine and rehabilitation physician, 1 geriatrician, 2 orthopedic surgeons, 5 physical therapists) and from both private and public practice.

To be eligible for the study, patients had to be in physical therapy programs and experience pain during at least one session. We aimed for diversification of the patient sample according to the following criterion: age (< 40 years old, n = 4; between 40 and 60, n = 4; > 60, n = 4), gender (7 women, 5 men), and place where the physical therapy program was performed (public practice, n = 6; private practice, n = 4; both, n = 2).

#### Recruitment

The patients were selected from the files of care providers identifying patients who had experienced pain during a physical therapy program. The care providers were recruited from the files of the members of the study board (pain specialists, GPs, physical medicine and rehabilitation physicians, rheumatologists, sociologists, anthropologists).

#### Interview protocols

After interviewing experts in the field of pain management and physical therapy and considering patient perspectives of pain induced by care management, we formulated a semi-structured interview protocol with open-ended questions. All interview protocols were as similar as possible so as to observe the range of variation of discourses and practices between medical experts and patients and to vary data sources to allow a deeper understanding of representations and practices linked to PIEM.

The interview protocols were structured by combining a "funnel-shaped" structure and the "itinerary method" [33-36]. The funnel-shaped structure was adopted to ensure that the interviews allowed for an inductive comprehension of the social reality at stake underlying the PIEM situation. The itinerary method of data collection was derived from anthropological data collection techniques and focused on objects, practices and the decision-making process. Applied to a therapeutic situation, it allows the researcher to follow the course of the patients, from the appearance of the pathologic condition, sometimes long before the physical therapy sessions, to the time of the interview, thus placing the PIEM in a broader context than the medical one. The postulate underlying this framework is that studying patient expectations of PIEM management cannot be limited to collecting explicit expectations that they might explicitly express: their expectations have to be identified throughout an analysis of the global social situation in which the PIEM occurred and was (or was not) managed, identifying contradictions, ambivalence, implicit expectations, and unanswered needs.

The interview protocols were planned as a loose list of themes and questions, interviewers continually adjusting their questions to the specific leads of the interview and pursuing unpredictable emergent data. They combined a thematic structure with chronological sequences to detail the therapeutic journey and the course of consultation. We tried to keep a similar path for all interviewees for comparison. Because these topic guides include open-ended questions, they could be presented to all interviewees, even if they relied on different individual experiences and decision processes. Of course, some chronological sequences could not be probed to the same extent with all interviewees. Example situations were for the patient journey sequence before the first physical therapy session, because physical therapists have limited insight into this part of the patient journey, and physical therapy sessions themselves, because physicians did not attend them. However, even if some interviewees had no experience with some topics, they usually had views, beliefs, or expectations about the topics, and we collected these data. To gain insights into the topic under study, we were careful to combine different types of data and collect not just opinions, beliefs, and expectations but also descriptions of effective personal practices and the contexts in which they took place.

The patient interview guide was designed to collect data on the following:

- The therapeutic journey, from the initial health problem to the physical therapy prescription and its organization. This gave the interviewers the opportunity to gather views and beliefs on pain and to identify and characterize previous pain experiences.

- Physical therapy sessions including exercise and mobilization; their description; PIEM appearance and patients' reactions and ways of expressing this pain; effects on the patient and their interpretation of this pain; and adjustments made in response to pain. This section allowed us to probe the patient-care provider relationship and communication and their views on physical therapy.

- Patient expectations related to the management of PIEM, particularly the assessment of PIEM, its prevention and treatment.

The physician interview guide was designed to collect information on the following:

- The physical therapy prescription process, and knowledge and views on physical therapy, specifically mobilization.

- Pain induced by the exercise and mobilization prescribed. Physicians were asked to express their views on PIEM, allowing us to probe their definition of this phenomenon and the process of interpretation they relied on, including the assessment process, if one existed; the effects on the patient and care providers; how physicians were engaged in the management of this pain; prevention and treatment practices; physician views about the treatment options available; and the description of the patient-physician relationship and the physician-physical therapist relationship and process of communication.

- Physician expectations and views of patient expectations.

The physical therapist interview guide was designed to study the following:

- The physical therapy session, in particular how prescriptions are filled and what indications are given by physicians; the decision process that governs the choice of physical therapy techniques; therapists' definitions of "mobilization techniques," how these techniques are used during the session, for what purposes, and how they are performed; the patient-therapist relationship, topics discussed, information given, and patient behaviours and demands.

- PIEM and more specifically the therapist definition of PIEM and comparison with other types of pain; PIEM identification and how therapists decipher patient behaviors; PIEM assessment; PIEM effects on treatment, on patients' life, on disease; the adjustments required and the therapists' practice to relieve and prevent PIEM; and views on treatment options available. This section allowed us to probe the patient-therapist and physician-therapist relationships and procedures of communication.

- Physical therapist expectations and views of patient expectations.

The mean duration of these interviews was 1½ hours for patients and 1 hour for care providers.

##### Procedures

The interviews were conducted during fall 2009. All patients were interviewed at home by trained sociologists. Care provider interviews took place where they practiced.

##### Analysis

The conversations were recorded digitally, transcribed literally, and analyzed by 2 different researchers (both sociologists) who also conducted the interviews. A third socio-anthropologist (DD) validated the coding and the analysis but did not take part in the interview process. A categorizing system based on the interviews was established. This index was consequently modified, categories and subcategories added as they emerged from the thematic content analysis. In a collaborative process, researchers continually checked that they had a common understanding of the categories generated. Numerous free categories were developed from the transcripts; these were discussed, adjusted and grouped in an iterative and inductive process. All the data were coded according to the final thematic index generated, and anonymity was respected.

## Results

### Presentation/characterization of the sample

The patient sample exhibited a diversity of professional activities (working, n = 5; retired, n = 3; unable to work, n = 4) and residence (8 urban; 4 rural). It also allowed for examining various medical situations (neck or back pain: 3 disk herniations, 1 spinal stenosis; knee pain: 1 hemarthrosis, 1 patella fracture; hip pain: 1 hip osteoarthritis, 1 hip replacement; shoulder pain: 1 scapula fracture, 1 humeral head necrosis; 1 ankle strain; trauma: 1 multiple fractures and burns).

The care providers' sample also ensured diversity of professional contexts, institutional status (6 were in exclusive private practice, 6 exclusive public practice, 2 both) and gender (6 women).

#### PIEM viewed by patients

Patients considered that PIEM during physical therapy programs is only one facet of a more general problem of pain induced by care (medical examination, administered personal care, treatments or investigations). They differentiated pain induced intentionally by care providers for diagnosis and that induced by care:

"The physician examined me from all angles. He pressed hard where it hurt a lot.... I don't blame him, it's his job." (Female patient, 24 years old, Houilles, received physiotherapy in secondary care hospital)

"One night, nurses came because I wet my bed. They tossed me just like a pancake to change the sheets. They hurt me so much that I screamed." (Female patient, 68 years old, Paris suburb, hip replacement, physiotherapy during hospital stay)

The description of PIEM was polymorphic and experienced as a degradation of HRQoL. The global therapeutic context affects this experience. PIEM could be sensed as "*an extra pain*" or as "*too much pain*" depending on the context, the impact of the patients' health condition in their everyday life, and their views on physical therapy. These factors may amplify or minimize the perception of PIEM. The occurrence of pain during a therapeutic program was cognitive dissonance for patients and a paradox, opposing the hope of getting better and the feeling of pain.

However, the objective of the care-induced pain was a pivotal element in how patients sensed pain and experienced it. It determined the interpretation, significance, and acceptance of the situation. Therefore, PIEM can be considered to be more a social construction than a simple painful sensation or a physiological response. It is indeed a combination of different components: sensorial, emotional, and social. The social dimension involves the meaning given to pain and the contexts of interaction in which pain is expressed.

The significance that patients attributed to PIEM was ambivalent; PIEM could be both positive and negative for them. Among positive representations of PIEM, patients cited interpretations such as "*part of a scarring or healing process*", "*a necessity in order to achieve recovery*", "*a useful alert to reconsider diagnosis or treatment*", or a protective signal, an indication of the "limits" beyond which one must not go. PIEM was not always seen as constructive. Although some individuals interpreted it positively, it still remained an undesired experience:

"I don't think it is a punishment, and I don't think it's a way to transcend oneself. Pain is pain! (...) I don't need pain to get mature or to grow internally.... If it can be avoided, so much the better." (Male patient, 25 years old, Paris, hemarthrosis, physiotherapy during hospital stay)

PIEM could also be interpreted negatively, as care providers' lack of technical competency or lack of attention and thoughtfulness:

"The day after my surgery, a physical therapist came. She was quite inhuman. I have a terrible memory of her. I had surgery on my right side and she grabbed my legs on the left side. Of course, I screamed!" (Female patient, 68 years old, Paris suburb, hip replacement, physiotherapy during hospital stay)

PIEM was referred to as control: when interpreted positively, it was pain "under control," and when viewed negatively, it was an unsought experience, associated with fear and helplessness:

"This pain (PIEM) is mostly coupled with a feeling of helplessness. It's frustrating because you don't know what ultimately comes from the pain and what comes from your feeling diminished." (Female patient, 26 years old, Châteauroux, surgery for hip fracture, physiotherapy at an outpatient clinic)

The impact of PIEM was complex, ranging from denial of symptoms to disruption of the therapeutic contract. When confronted with PIEM, patients did not systematically express their pain. Showing and verbalizing pain was not a neutral decision, and it could be viewed as a social and strategic decision. Strategies for emphasizing, softening, minimizing, or "silencing" were observed, depending on the sense given to the physical therapy, the pain felt, and the social context of occurrence:

"Maybe I should have accepted more [than I did], or perhaps I accepted too much. (Is it difficult to judge?) Yes. It's like for syrups: before they were bitter but (to get better you had to take them anyway. Physical therapy is the same! It's not because it hurts you that you have to stop. Because that's what makes you better, so I do accept a little bit. But when it hurts too much, I stop.... (Male patient, 36 years old, Ocquerre, patella fracture, physiotherapy at an outpatient clinic)

PIEM expression appeared to be a real issue for patients who tried to control it. Shouting might be felt as a loss of control, an inadequate verbalization, or a worthless option:

"I wasn't screaming. A kid might have screamed reacting to this kind of pain but not an adult: I just gritted my teeth." (Male patient, 25 years old)

"There are people who scream right away, but you shouldn't -- otherwise you can't progress.... At physiotherapy, they told me that I no longer feel the pain.... I do feel it, but I never say anything because I know that it [the physical therapy] must be done.." (Female patient, 61 years old, Boulogne-Billancourt, trauma during car crash, physiotherapy during hospital stay and at an outpatient clinic)

Expressing one's pain could be felt as socially incongruous, and patients adjusted their expression according to how they defined the PIEM and how they interpreted their social situation:

"Last week, I had a crying fit at the hospital. I couldn't stop crying. That happens regularly. And yet, I wasn't off worse than before, and the worst thing is that I would hide the crying because I was in the physiotherapy room and I was afraid of being watched." (Female patient, 61 years old, Boulogne-Billancourt, trauma during car crash, physiotherapy during hospital stay and at an outpatient clinic)

The "presentation" of PIEM also depended on bystanders. For instance, one patient was allowed to express her pain by singing, because shouting was not an option for her in a care context; others limited themselves to non-verbal indicators:

"During the [physical therapy] session it hurt. I was sweating [with pain]." (Male patient, 77 years old, Villiers-le-Bâcle knee osteoarthtritis and low back pain, physiotherapy during hospital stay)

Interactions with the health care providers affected patients' response to PIEM, sometimes encouraging patients to bear the pain and the physical therapy at the same time:

"They [physical therapists] are so nice. They're working for us but they are so involved that you also want to do it for them." (Female patient, 68 years old, Meudon, hip replacement, physiotherapy during hospital stay)

In some aspects, the acceptance or minimization of the expression of PIEM may be part of a specific exchange between patients and physical therapists, denoting reciprocity in the care process. The information delivered to patients was also an active part of this relationship and could have an influence:

"I remember a physical therapist who told me: "we're not going to relieve you but we are going to teach you how to live with your pain". It made me think..... If they hadn't told me at the rehabilitation center to do things but to do them reasonably, I think I would have stopped immediately, saying that this pain was abnormal." (Female patient, 43 years old, disk herniation, physiotherapy during hospital stay and at an outpatient clinic)

Nevertheless, the occurrence of pain during a physical therapy session had negative consequences for the patient and the therapeutic contract. Patients expressed feelings of loss of confidence with the medical management of the situation and fear of reactivation of the disease:

"For a while it did not hurt anymore. I began to recover. But the physical therapy, the manipulations, it woke up the pain. What bothered me most was that it (my ankle) had begun not to hurt, but the physiotherapy sessions brought back the pain. That's all I noticed." (Male patient, 46 years old, ankle strain, physiotherapy at an outpatient clinic)

#### PIEM viewed by care providers

Care providers agreed that PIEM is generally not a part of their management strategies and that a generally accepted definition is lacking. When analysing the meanings of PIEM, care providers emphasized the positive dimensions of PIEM, such as pain as a "red flag" allowing for treatment adaptations or as a signal of evolution of the underlying pathology:

"Pain is a limiting factor; it is an alarm system that tells you not to go further. For instance, if you consider hand surgery, it is useless to prescribe painkillers to relieve the pain, because there are things you mustn't do (and pain will stop you doing them)." (GP, private practice)

"(I view PIEM differently according to the situation). It is not the same the week after the surgery as it is six weeks afterwards, when normally the pain is decreasing [and] the patient regains mobility. If the pain or the level is reversed at this time, it means that there is a problem, a complication. It can be a hematoma, an infection, or dislocation of the orthopedic material. (Orthopedic surgeon, public practice)

These "virtues" seemed to have more weight than the negative dimensions of the situation. Care providers' downplaying the negative dimensions of PIEM involved a double process: the purpose of PIEM (patients suffer to get better) and the reminder of medical deontology and the absence of intentionality ("we do not do it on purpose"):

"When you examine a patient, you may cause pain. There is no malice intended. It is because it helps us make a diagnosis. It's the same for physical therapy: we might actually have to cause pain. We are not trying to hurt the patient; it's to make them better."(Rheumatologist, public and private practice)

"In my mind, pain is impossible in physical therapy. It may hurt at the time but afterwards, it is so much better that it is a 'blessing in disguise'. That's why it does not even occur to me to anticipate [the pain]. I am probably wrong, but that's the way I think automatically." (GP, private practice)

PIEM was identified mainly by physical therapists and less by physicians. Different modalities of pain expression were reported: clinical signs, attitudes, and verbalization. The identification of PIEM did not imply management of PIEM; the patient and physical therapist discussed the level of pain and what was acceptable to the patient before treatment might be modified.

Although several care providers tended to minimize the consequences of PIEM, consequences cited were a worsening of the patient's clinical situation, fears about physical therapy, rejection of the physical therapist, and refusal of care:

"I think if it's too painful, it will have psychological consequences in following up the physical therapy program. Patients will be afraid to come. They will look on physical therapy as something horrible instead of thinking it will improve (their condition)." (GP, private practice)

"If you exceed the limit once, the patient may no longer want to see you. It's rare but it happens. If you simply exceed the limit once and the pain is unbearable, it's all over with the patient." (Physical therapist, public practice)

#### PIEM management

Care providers admitted the existence of PIEM, but stated that it had little or no impact on their practice for managing patients' condition as compared with side effects observed with other treatments.

##### Assessment

Physical therapists were the only care providers to assess PIEM; they mainly used standardized tools such as visual analog or numeric scales, but the use of pain scales was not systematic. Physicians did not assess PIEM and had limited confidence with tools used to assess pain in general:

"There are paradoxical situations: there are patients who arrive smiling but say their pain level is 10. The scale is a very specific tool. It is a valuable tool.... I stopped using those scales because of that, because it meaningless for patients." (GP, private practice)

Patients recognized that pain levels are regularly assessed but had ambivalent views concerning the tools used for these evaluations, especially scales. Some patients considered these evaluations important for the care provider's comprehension of their clinical situation, whereas others questioned their use.

"It [pain scale] is probably useful for doctors in order to correct things. They do need benchmarks. It allows them to know how people suffer." (Male patient, 57 years old, Paris, hip osteoarthritis, physiotherapy during hospital stay in a tertiary care teaching hospital)." The poor [care providers]! Otherwise, how can they assess [pain]?" (Female patient, 26 years old, Châteauroux, surgery for hip fracture, physiotherapy at an outpatient clinic) "It is required for the hospital service but for patients it's not very useful." (Female patient, 43 years old, Paris, disk herniation, physiotherapy during hospital stay and at an outpatient clinic). "They give you a scale and ask you to say how much pain you are feeling on a scale of 0 to 10. I refuse to rate it with a number because I can't. It can always hurt more than you think it could." (Female patient, 63 years old, Paris, humeral head osteonecrosis, physiotherapy during hospital stay)

##### Treatments

Few standardized procedures exist to prevent or treat PIEM. Physical therapists, the first-line care providers dealing with PIEM, have limited treatment options. Responses to the perceived or expressed complaints include modulating the perception of pain (distraction, encouragement, explaining the purpose of the treatment) and pain inducer (modifying the rhythm or type of exercise, ending the exercise session), and using pain-oriented techniques (e.g., massage, transcutaneous electrical neural stimulation).

Treatment of PIEM was subordinate to general pain management for chronic situations or for post-surgical periods. Specific treatment for PIEM was prescribed infrequently. Pharmacological and nonpharmacological options for PIEM related to the general treatment of the underlying clinical situation (chronic diseases or post-surgical periods). Thus, PIEM treatment depended on physicians' choices for general pain management, but patient impressions of pain killers also influenced PIEM treatments:

"I avoid drugs; you know they're not good. If you take a paracetamol as soon as you have a headache, sooner or later your body will adapt to it, and instead of 500 mg, it will ask for more... The way I see it, you could say that it's part of our diet.... I am not happy when I leave the drugstore with a huge bag!" (Male patient, 36 years old, Ocquerre, patella fracture, physiotherapy at an outpatient clinic)

"Morphine terrifies me. Even the term is terrible! A light death, a small death, an adorable death." (Female patient, 63 years old, Paris, humeral head osteonecrosis, physiotherapy during hospital stay)

These impressions were organized around central themes:

- toxicity

"On one hand, drugs mess you up you, and on the other, they treat you. I am sure of that... but I do look at the leaflets in order to see if I'm not going to be poisoned." (Female patient, 63 years old, Paris, humeral head osteonecrosis, physiotherapy during hospital stay). "(How do your patients view antalgics?) They think they're harmful in the long run. They say they don't want to take them anymore.." (Physical therapist, private and public practice)

- fear of loss of efficacy with time

"And you get used to drugs; your blood must get used to them, because before, they had more effect on me." (Male patient, 77 years old, Villiers-le-Bâcle, knee osteoarthritis and low back pain, physiotherapy during hospital stay)

- perception of lack of efficacy of over-the-counter pain medications

"(Did they give you any sort of treatment along with it?) No, I didn't get anything. (You say you're taking paracetamol?) Ah, if that's what you mean by treatment, well then yes: I'm taking paracetamol but it's not doing anything for me." (Male patient, 57 years old, Paris, hip osteoarthtritis, physiotherapy during hospital stay in a tertiary care teaching hospital)

- and severe side effects with opioids:

*"They asked me if I wanted morphine... it took me 8 days to get my intestinal tract back in order." (Female patient, 68 years old, Meudon, hip replacement, physiotherapy during hospital stay)*.

"It made me nauseous though; it caused vomiting." (Female patient, 61 years old, Boulogne-Billancourt, trauma during car crash, physiotherapy during hospital stay and at an outpatient clinic)

The negative views of pain killers influence compliance:

"I am a bit anti-medicine, so the less I take, the better I feel." (Male patient, 25 years old, Paris, hemarthrosis, physiotherapy during hospital stay)

Compliance with prescribed treatments also related to dosage adapted to pain level, occurence of side effects, and perceived efficacy.

GPs did not mention the possibility of PIEM, and only some rehabilitation units in tertiary care hospitals proposed standardized procedures.

##### PIEM prevention

We observed three different types of practices concerning PIEM prevention. One was the absence of any consideration of this topic by GPs, who stated that they did not consider the situation, and by physiotherapists, who stated that they did not induce pain with the techniques they use:

"(If we focus on physiotherapy, do you think that it might hurt?) I never tell my patients that physiotherapy can hurt simply because I don't think of it. Moreover, I don't know whether it will hurt or not." (General practitioner, Paris)

"During the session, I try to stop before pain occurs or to fight against pain. My technique is to be of benefit, to relieve, not to work through pain.... The first thing you learn in your studies when you are a physiotherapist is to work without provoking pain. That said, there are some big brutes, fewer and fewer I think.... who force even when it hurts... but for me, there's no point in that." (Physiotherapist, Paris)

Another practice identified is selective PIEM prevention. Prevention is not systematic ("*from time to time*") and not standardized. It concerned patients who feared physical therapy, with specific painful situations, such as with adhesive capsulitis or certain surgeries that require early exercise:

"(Do you prescribe something to prevent pain during physiotherapy sessions?) Sometimes I warn my patients. I tell them to take a painkiller one hour before the session or an anxiolitic to relax. It is because I am an old physician.." (GP, Paris)

"(Is there any specific warning, such as 'I know that the therapy is going to hurt, so I apply a pain killer before or just after the session'?) We do it from time to time. Again, our treatments are really individualized. If we think that the therapy is going to be painful, we give painkillers with a specific schedule." (Geriatrician, Hospital, Paris suburb)

*"(Do you make these prescriptions to *anticipate *or to *treat *the pain induced by physiotherapy?) It depends. Some exercise programmes are more painful than others. If I take my last patient, whom I've treated for shoulder pain, I did prescribe morphine. She was close to adhesive capsulitis...I had to act quickly to free the shoulder, and I told her to take pain killers, in this case morphine." (GP, Villers-Cotterêts, north of France)*

The third practice was a standardized protocol of prevention proposed by some GPs and in some rehabilitation units:

"When I prescribe physiotherapy, I also prescribe analgesics. I explain to the patient that he may experience pain and that he will have to take his treatment. I also prescribe non-steroidal anti-inflammatory drugs. I tell my patients that it is temporary, that it is for 2 or 3 days when it is most painful.. I also explain to them that they should tell the physiotherapist when it hurts." (GP, Villers-Cotterêts, north of France)

The presence or absence of PIEM prevention procedures seemed to depend less on the status of the institution of the care provider and more on the sensitivity of the care provider to pain management.

##### Coordination of care and follow-up

Poor coordination of care is a factor that may worsen PIEM management. Multiple care providers are involved in PIEM management and follow-up. Nurses, physiotherapists, surgeons, anesthetists, and specialists may be involved in the care of inpatients, with only primary care physicians and physiotherapists involved in the care of outpatients. For inpatients as well as outpatients, PIEM follow-up was characterized by poor transmission of information, in part because of professional compartmentalization:

"(How does communication with physicians work?) In surgery units, it is very difficult. If it's urgent, we phone; otherwise we look for them in the corridors, we run after them. It's not very easy, but that's how it is; you can lose a day of treatment for example." (Physiotherapist, tertiary care hospital, Paris)

"When we arrive in the morning, we discuss patients with nurses to schedule our intervention between dressing changes, and we manage to reschedule physiotherapy according to analgesics prescription (Is that easy to do?) No, it's not always easy. You have to be organized." (Physiotherapist, tertiary care hospital, Paris)

A lack of human resources was also mentioned:

*"We have analyzed our practice for pain management after total knee replacement. We realized that we were not efficient and that our pain management was not satisfactory. (Why?) I think that it is a matter of number of physicians. Here there is one and a half physician for 75 patients. The physiotherapists do not always find us." (Physical medicine and rehabilitation physician, Hospital, Issy les Moulineaux, Paris suburb)*.

Information about PIEM was transmitted mainly in rehabilitation units, but it might be partial or delayed. For outpatients, there was practically no transmission of information between GPs and physiotherapists, except for situations of significant medical complications.

#### Patient expectations about PIEM management and treatments

Patient expectations about PIEM management included more information and transparency from care providers about what factors induced pain, how to assess pain, and whether it requires a specific treatment:

"There is no debriefing on this.... The first day and the last day, we fill in a little questionnaire for the physician to assess the impact of pain on our everyday activities. We fill it in, but it's the same, we don't get any feedback. I would have liked it, for example, if someone had told us about our progress after the sessions" (Female patient, 43 years old, Paris, disk herniation, physiotherapy during hospital stay and at an outpatient clinic)

They also expected education on how to better prevent stress and anxiety generated by pain, counseling on how to anticipate PIEM and on mobilization, and accurate adaptations of physical therapy programs according to pain intensity. These expectations were modulated by patients' own interpretations and behaviors concerning pain in general and PIEM. Some patients were fatalistic about PIEM:

"I think it's better to do the physiotherapy session without medication. That way if there is a problem you can feel it. If it hurts, it is better to take analgesics after pain occurs, because if you take it before and there is a problem, you won't feel it." (Male patient, 46 years old, Paris, ankle strain, physiotherapy at an outpatient clinic)

*"In any case, even at home, I can't control pain, and I don't see how I could do so during physiotherapy sessions." (Female patient, 43 years old, Paris, low back pain, physiotherapy at an outpatient clinic)*.

Not all patients suggested that care providers should be more involved in PIEM recognition and management.

Expectations about pain killers were heterogeneous. The criteria mentioned were efficacy, rapidity of action, absence of side effects, and methods of administration. For over-the-counter drugs and acetaminophen, expectations were mainly for more efficacy, whereas for opioids, they were mainly for fewer side effects:

*"Paracetamol. It might be worse if I didn't take it, but it is totally inefficient" ( Male patient, 57 years old, Paris, hip osteoarthritis, physiotherapy during hospital stay in a tertiary care teaching hospital)*.

"I had a lot of pain killers. Paracetamol and codeine didn't do any good. When I was on these prescriptions, sometimes I didn't take anything because I figured 'if that's what a pain reliever is...' I don't take medicine if it does nothing for me." (Female patient, 43 years old, Paris, disk herniation, physiotherapy during hospital stay and at an outpatient clinic)

"I couldn't stand tramadol. I was dizzy, I threw up, and I didn't even know my name anymore." (Female patient, 24 years old, Houilles, physiotherapy in secondary care hospital)

"I felt nauseous, I even threw up, and the last time I took tramadol, I took 2 tablets and I was completely drowsy, really, really sleepy but when I was taking 6 tablets a day, I didn't experience those side effects." (Female patient, 43 years old, Paris, disk herniation, physiotherapy in an outpatient clinic and in a tertiary care hospital)

#### Care provider expectations about PIEM management and treatments

Care providers had few expectations about PIEM management or specific continuing education programs on PIEM, either because they were satisfied with actual practice or not sensitized to PIEM. They did express a need for tools or procedures to better identify, assess, and follow PIEM:

"I don't really know how to measure the pain... It is just like these super PAS scales from 0 to 10, but you'll never manage to get an orthopaedic surgeon to keep this thing in his pocket. Even my goniometer, that I use every day, I can't keep it in my pocket." (Orthopaedic surgeon, Paris, tertiary care hospital)

"For pain assessment, there have been policies that were widely circulated that might give you a false good conscience, saying 'I've used my little EVA scale, so there, there's no problem'". (Geriatrician, Issy Les Moulineaux, Paris suburb, secondary care hospital)

Care providers' opinions about pain killers strongly depended on the images reflected and opinions expressed by patients, and the criteria that they mentioned were similar to those defined by patients.

As for continuing medical education programs, care providers mentioned the already large number of educational programs and their limited time to participate in these programs:

"We are so overwhelmed. We choose one or two and that's it. It is impossible to manage if you get in any deeper." (Orthopaedic surgeon, tertiary care hospital, Paris)

Several physicians pointed out the need for a large sensitization campaign on pain before proposing specific educational programs on PIEM. Physicians expressed a need for better education on the pharmacokinetics of analgesics and on prescriptions for physiotherapy:

"My expectation would be to have explanations on drug therapies for pain, their side effects, those I can associate. I'm missing that. I receive physician visitors from the industry, but that's not enough." (Physical medicine and rehabilitation physician, Issy les Moulineaux, Paris suburb, secondary care hospital)

Education on the patient-physician relationship concerning pain management was also mentioned:

"(If you should have an education program on 'pain and mobilization', what format would be most appropriate?) I think the psycho-behavioural approach is important. It's important for almost everyone. The relationship you have with the patient has a great influence on pain. A relationship of trust can help them forget about pain and concentrate on movement." (Physiotherapist, outpatient clinic, Villiers le Bel, Paris suburb)

Physicians insisted on the need for educational programs aimed at increasing competency rather than knowledge and on interdisciplinary programs because PIEM management implies different and complementary skills.

## Discussion

To our knowledge, this is the first qualitative study of patient and care provider views of PIEM. Our results suggest an important lack of knowledge and recognition of this situation by care providers not directly involved in physical training or physiotherapy programs.

Even though patients had ambivalent and heterogeneous views about PIEM, the occurrence of PIEM can be considered a serious adverse event because of its negative consequences on patients and on their therapeutic contract with care providers, which can lead them to suspend or end treatment, lose confidence in medical management, and return to the condition of chronic pain.

Care provider views of PIEM differed from those of patients. PIEM was sometimes viewed negatively due to lack of competency of a single and isolated professional and therefore a relatively infrequent situation or to particular situations with patients highly vulnerable to pain. In the latter case, the PIEM importance was minimized, and some care providers indicated that the risk of PIEM was more that it would slow recovery than slow the treatment process.

Our results also suggest that PIEM assessment is far from optimal because of care provider low confidence in assessment tools and patient ambivalent views of the usefulness of assessment. One barrier to the assessment of PIEM is patient lack of comprehension of the therapeutic objectives of this assessment, which sometimes leads to passive submission behaviors regarding PIEM assessment and can in turn be harmful to the quality of the assessment. The process of evaluation could be improved by recognizing its utility for therapeutic decisions, improving the quality of the patient-physician relationship, clarifying its use to patients, and proposing more relevant assessment tools to patients and care providers.

Our results reveal several barriers to specific PIEM treatments. Physiotherapists are care providers who frequently deal with PIEM. Although they have access to non pharmacological options such as physical and cognitive techniques, education received on this topic is at least limited. Cooperation among care providers is needed to efficiently deal with this clinical situation. However, lack of communication among care providers seemed greater than the existence of structured protocols to prevent or treat PIEM. In addition, by nature, the physiotherapist's work depends on patient cooperation, and one of the treatment options available is to modulate factors inducing pain, which leads patients to discontinue mobilization exercises. Another barrier is patient and physician global negative view of pain killers. We also observed these negative views of pain killers among French patients with knee osteoarthritis [37]. The trivialization of the effects of acetaminophen or paracetamol, for example, leads patients to consider that these are not real treatment options and they thus become less cooperative with treatment. In this case, patients' views of acetaminophen and paracetamol do not match the scientific and pharmaceutical definitions of these agents and are reinterpreted according to patients' cultural and social systems. PIEM pharmacological treatment might be facilitated by specific patient education and counseling on pain killers, particularly acetaminophen or paracetamol, to increase patients' adapted self-management of these medications. Self-management of pain killers is already recommended for low-back pain [38] and knee OA [39].

To help physical therapists manage the emotional and cognitive aspects of PIEM, educational programs during their initial education or later continuing education could be proposed. In addition, educational programs for physicians should increase their knowledge of non pharmacological options for management of PIEM which should lead to propose cognitive and coping strategies more often to better manage PIEM. Similar barriers [40] and potential facilitators [41] have been suggested by qualitative studies analyzing management of low-back pain in the workplace.

Some obstacles to PIEM prevention are organizational constraints, such as the lack of human resources, mainly in institutions, and care planning, such as communication among nurses, physicians, and physiotherapists. PIEM prevention could be facilitated by better organization of medical care (prescription and follow-up), providing patients with specific educational programs about cognitive and coping strategies to alleviate pain and about painkillers and their use, and care provider training in non pharmacological treatment options and the pharmacokinetics of analgesics. PIEM follow-up is characterized by poor transmission of information among care providers, and one way to optimize this transmission might be the systematization of standardized information to be circulated among the patients and care providers involved in the process. However, this approach cannot be optimal without care providers being involved in the process, and facilitators for the adoption of these procedures have yet to be determined.

Patients' expectations concerning PIEM have two main aspects: global management and specific treatments. Expectations concerning managing PIEM are mainly related to the implications of treatment and more communication between patients and care providers. To be effective, a strategy aimed at optimizing this process would lead to a "win-win" situation for both patients and care providers. Expectations concerning specific treatments may appear unrealistic, which points to the need for more education and counseling.

The lack of care providers' expectations about PIEM is the result of the lack of knowledge about the situation and the tendency to minimize its impact on patients and on disease management. Their expectations mainly concern the need for formalized support for the identification, assessment, and management of PIEM. However, care providers did not spontaneously express a need for specific continuing medical education on this topic. When it was suggested to them by interviewers, care providers mentioned the large range of programs already available, and they insisted on the need for prior sensitization about the general management of pain and a pragmatic approach to increase their skills. Thus, increasing care providers' adherence to and use of education programs on this topic should emphasize the interest for both patients and care providers in better identifying and managing PIEM.

Our work has limitations. The first is that we restricted our investigation to a French context. How the health care system is organized and the cultural context in France probably affect views and expectations in this field. Transcultural qualitative studies are needed to address this question. Moreover, we did not search for non-confirming cases. Another limitation is our rather small sample of patients and practitioners. Therefore, we cannot exclude that this study might under represent the range of views that patients and care providers have of PIEM. However, we tried to stratify this sample to reflect diversity, as recommended for qualitative studies expecting a range of representations, practices, interactions, and expectations of a subject [42].

Finally, this work underlines the views that patients and care providers have of PIEM, which limit the management of PIEM more than the material constraints or the social dynamics of the patient-care provider relationship. Ethnographical observations are necessary to probe the logic underlying these relationships and detail potential value conflicts [43].

## Conclusions

Patients and care providers have differing views of PIEM in the overall management of disease. PIEM management could be optimized by sensitizing care providers to the situation; improving communication among care providers; providing information and education to patients and care providers to recognize, assess, prevent and treat PIEM; and elaborating and proposing standardized structured procedures to manage this particular clinical situation. The appropriation, mobilization, and efficacy of information and education for care providers and patients need further investigation.

## Competing interests

Financial competing interests

This work was funded by Bristol-Myers Squibb. SA, DD, ASB and SP received honoraria from BMS. EB is employed by BMS.

There is no other competing financial interest

Non-financial competing interests

The author declares that they have no competing interests.

## Authors' contributions

SA, DD, MMLC, EB, FR, and SP contributed to Conception and design of the semi-structured interview study, SA and ASB to data collection, SA, DD, ASB, and SP to data analysis, SA, MMLC, and SP to drafting the manuscript, DD, ASB, FR, and EB to critical review of the manuscript. All authors read and approved the final manuscript.

## Endnotes

^1 ^Phonetically, in French, the word "morphine" sounds like "death" (*mort*) and (*fine*), which means "fine", "exquisite", "thin", "small" and led the patient to associate "morphine" with "a little death". Etymologically, the word refers to the Greek name for the God of Dreams, Morpheus.

## Pre-publication history

The pre-publication history for this paper can be accessed here:

http://www.biomedcentral.com/1471-2474/12/172/prepub
